# The Negatively Charged Regions of Lactoferrin Binding Protein B, an Adaptation against Anti-Microbial Peptides

**DOI:** 10.1371/journal.pone.0086243

**Published:** 2014-01-20

**Authors:** Ari Morgenthau, Amanda Beddek, Anthony B. Schryvers

**Affiliations:** Department of Microbiology and Infectious Diseases, University of Calgary, Calgary, Alberta, Canada; Columbia University, United States of America

## Abstract

Lactoferrin binding protein B (LbpB) is a bi-lobed membrane bound lipoprotein that is part of the lactoferrin receptor complex in a variety of Gram-negative pathogens. Despite high sequence diversity among LbpBs from various strains and species, a cluster of negatively charged amino acids is invariably present in the protein’s C-terminal lobe in all species except *Moraxella bovis*. The function of LbpB in iron acquisition has yet to be experimentally demonstrated, whereas *in vitro* studies have shown that LbpB confers protection against lactoferricin, a short cationic antimicrobial peptide released from the N- terminus of lactoferrin. In this study we demonstrate that the negatively charged regions can be removed from the *Neisseria meningitidis* LbpB without compromising stability, and this results in the inability of LbpB to protect against the bactericidal effects of lactoferricin. The release of LbpB from the cell surface by the autotransporter NalP reduces the protection against lactoferricin in the *in vitro* killing assay, attributed to removal of LbpB during washing steps, but is unlikely to have a similar impact *in vivo.* The protective effect of the negatively charged polysaccharide capsule in the killing assay was less than the protection conferred by LbpB, suggesting that LbpB plays a major role in protection against cationic antimicrobial peptides *in vivo.* The selective release of LbpB by NalP has been proposed to be a mechanism for evading the adaptive immune response, by reducing the antibody binding to the cell surface, but may also provide insights into the primary function of LbpB *in vivo.* Although TbpB and LbpB have been shown to be major targets of the human immune response, the selective release of LbpB suggests that unlike TbpB, LbpB may not be essential for iron acquisition, but important for protection against cationic antimicrobial peptides.

## Introduction

Lactoferrin, an 80 Kda bi-lobed host iron-binding glycoprotein, is found at varying levels throughout the body, the highest being at mucosal surfaces and sites of inflammation [1], [2]. Although a primary role of lactoferrin is to sequester available iron in the extracellular milieu, it has many other important functions that combat pathogens within the host (Reviewed in [3]). Lactoferrin is a major component of the secondary granules of neutrophils and is released in its iron free form at sites of inflammation resulting in high local concentrations. In addition to lactoferrin being able to remove freely available iron from its surroundings, it has been previously shown that apo-lactoferrin has antibacterial properties separate from iron sequestration [4]. Apo-lactoferrin, the form found in the granules of neutrophils, is a source of cationic antimicrobial peptides released by proteolysis from the N-terminal region of lactoferrin [5].

Lactoferrin receptors have been well characterized in pathogenic *Neisseria* spp. and their importance has been confirmed in a *Neisseria gonorrhoeae* infection model in humans [6]. The lactoferrin receptor is composed of an integral outer membrane protein, lactoferrin binding protein A (LbpA), and a surface-exposed membrane bound lipoprotein, lactoferrin binding protein B (LbpB) [7]. *In vitro* studies have shown that LbpA, which acts a membrane channel for iron removed from lactoferrin, is necessary for iron acquisition from lactoferrin [8], [9]. In contrast, the role of LbpB in iron acquisition has not been established. However, LbpB has been shown to confer protection against human lactoferricin [10]. The homology between transferrin binding protein B (TbpB) and LbpB has resulted in many inferences regarding the function of LbpB based on the known functions of TbpB. However LbpB differs from TbpB in multiple important aspects, particularly in the presence of negatively charged regions localized to the LbpB C-Lobe. The presence of negatively charged regions have previously been used to distinguish LbpB from TbpB in bioinformatics analyses [10].

LbpBs have been identified in a variety of Gram-negative pathogens and, with the exception of LbpB from *M. bovis*, all posses one or more clusters of negatively charged amino acids in the C-terminal lobe. Two thirds of LbpBs examined from *N. meningitidis* posses two negatively charged regions, one substantially larger than the other [11]. A variety of functions have been suggested for the negatively charged regions including them serving as a binding site for lactoferrin or functioning as an immunodominant epitope [12] [13]. LbpB is selectively released from the bacterial surface by NalP, which would enhance the immune evasion properties, as release of LbpB by NalP has been shown to reduce *N. meningitidis* susceptibility to anti-LbpB antibodies in serum bactericidal assays [14]. Recent analysis *of N. meningitidis* isolates before and after an accidental human passage found that the *nalP* gene was turned on and up-regulated during human passage, suggesting that the NalP-mediated protein release plays an important role during an infection [15]. This notion is supported by *ex vivo* whole blood infection models which found both *nalP* and *lbpB* genes to be up-regulated in human blood [16]. Although survival assays in whole blood with LbpB-ve mutants were not performed, the presence of NalP was shown to improve bacterial survival, suggesting that the release of proteins by NalP is important during infection of human blood [16].

In this study we explore the role of the negatively charged regions of LbpB in conferring protection against the antimicrobial peptide lactoferricin.

## Results

### Production of LbpB Derivatives Lacking the Negatively Charged Regions

**Figure 1:**
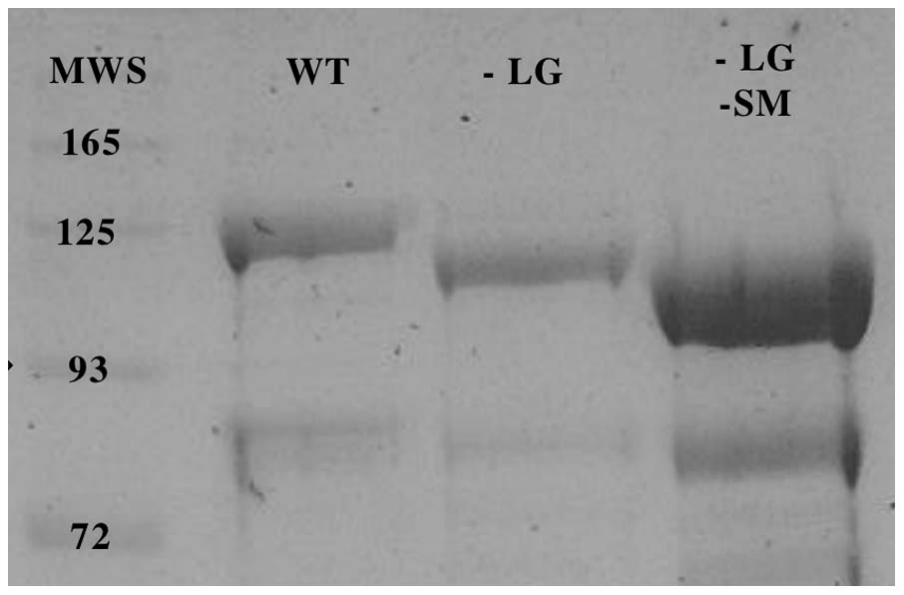
Production of mutant and wild-type LbpBs. Expression experiments were performed with plasmids carrying the gene encoding aa 54–737 of the mature wild-type LbpB (WT) from *N. meningitidis* strain MC58, a derivative with the large negatively charged region removed (−LG, aa 469–533) and a derivative with both the large and small negatively charged regions removed (−LG, aa 469–533; -SM, aa 691–721). The recombinant fusion proteins containing an N-terminal polyhistidine tag, a maltose binding protein and a tobacco etch virus protease cleavage were expressed in an *E. coli* T7 based expression system and isolated by a nickel NTA affinity resin. Purified samples normalized to equivalent quantities of the original culture volume were applied to an 8% polyacrylamide SDS-PAGE gel and stained for protein. The upper bands found around ∼125kDa represent the recombinant fusion proteins whereas the lower bands represent LbpB released from Mbp.

Based on the premise that the negatively charged regions in LbpB were not part of critical structural elements of the protein, we designed and tested mutant LbpBs with the negatively charged regions removed. The first step was to clone the region encoding the mature LbpB of *Neisseria meningitidis* strain MC58 (minus the C-terminal cysteine, aa 2 −737) into an vector designed for high-level expression in the cytoplasm of *Escherichia coli* [17]. The expression plasmid encoded an N-terminal maltose binding protein partner with polyhistidine tag and TEV protease cleavage site. Inverse PCR primers were designed to remove the large (aa 469–533) or small (aa 691–721) negatively charged regions and then various N-terminal truncations of the wild-type and mutant LbpBs were subcloned into the expression vector. Expression studies with the recombinant plasmids demonstrated that yields of the mutant proteins were equal to or better than that for the wild-type protein (Figure 1). After TEV cleavage and purification the mutant proteins remained stable and were used in crystallization trials for structural studies. These observations indicate that the negatively charged regions do not include core elements required for maintenance of the structure.

**Figure 2:**
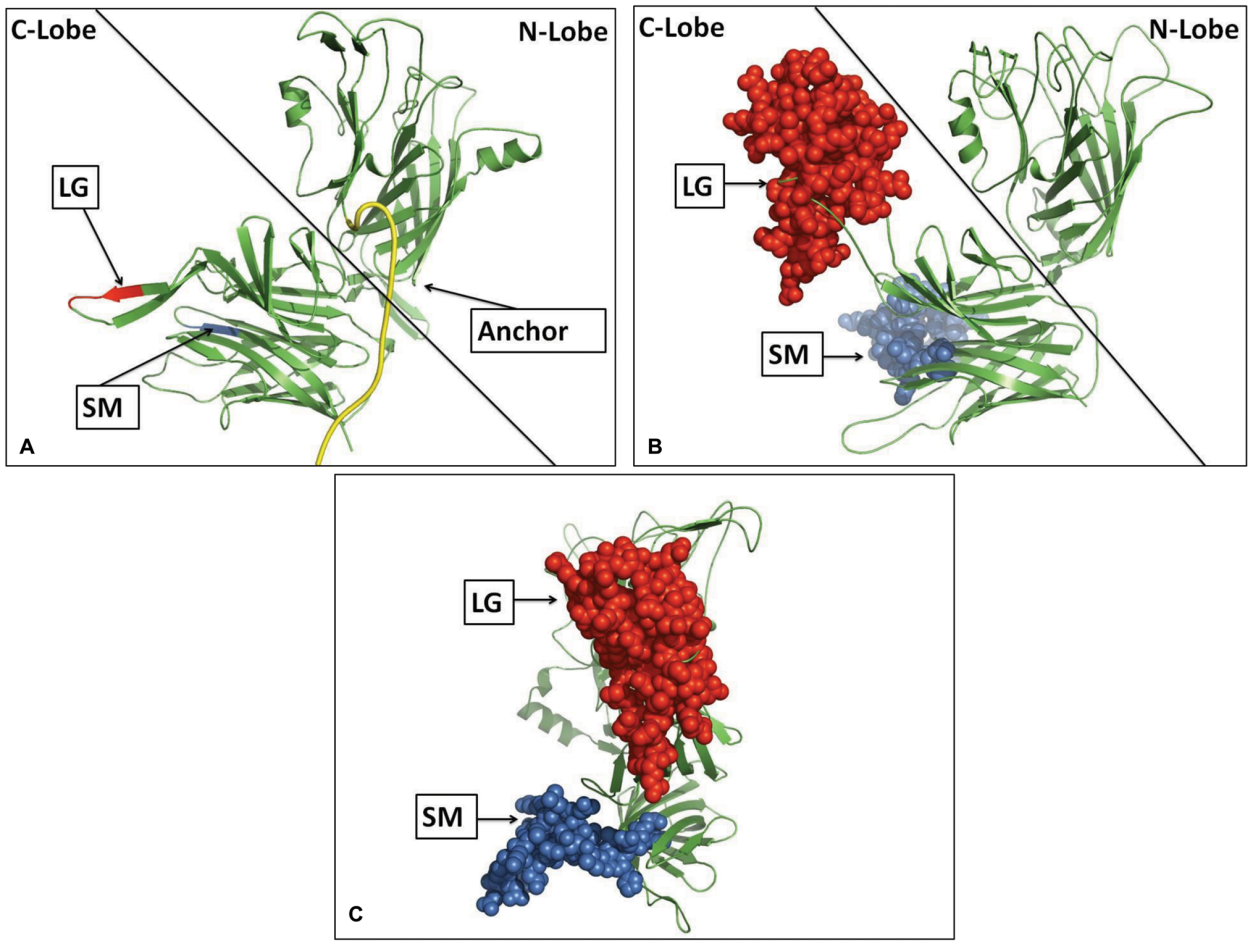
Structural model of a mutant and wildtype LbpB from *N. meningitidis* strain MC58 . Panel A illustrates a structural model of the mutant LbpB that lacks the negatively charged regions. The sites of insertion for the negatively charged regions are indicated in red (large, LG) and blue (small, SM). The model was generated using the Swiss-Model server using the known TbpB structure from *A. pleuropneumoniae* as a template. The anchor peptide region, which tethers the protein to the outer membrane and is cleaved by NalP, is highlighted in yellow. Note that this only includes 23 of the 46 amino acids that constitute the entire anchor peptide region (aa 23–46) of the mature protein. A structural model of the wild-type LbpB is shown in Panel B, illustrating the size of the negative charged regions. This structural model was generated using the structure in Panel A as a template. The large negatively charged region (LG) is shown in red while the small (SM) is shown in blue. In panel C the LbpB from panel B is displayed in “front view” orientation showing how the negatively charged regions can obscure the surface of the C-lobe ‘Cap’ region.

Due to greater homology and lack of large gaps in sequence alignments, the LbpB protein lacking both of the negatively-charged regions was more suitable for preparing a structural model based on known structures of TbpBs [18], [19]. Thus we prepared structural models for the mutant LbpB from MC58 lacking both negatively charged regions using either the TbpB from *Actinobacillus pleuropneumoniae* or TbpB from *N. meningitidis* M982 as a template. Both models predicted the same location for the negatively charged regions. We have illustrated the model generated with the *A. pleuropneumoniae* TbpB as template since it displayed more of the anchor peptide region (Figure 2, Panel A). The protein is comprised of two structurally similar lobes, each containing an N-terminal handle domain and a C-terminal beta-barrel domain. In this structural model the site of insertion for the large negatively charged region is found in the loop between β22 and β23 of the C-lobe handle domain whereas the small negatively charged region is found in the loop between β30 and β31 of the C-lobe barrel domain (numbers are based on the nomenclature in [20]). The locations for the insertion sites of the negatively charged regions are consistently obtained with models developed with *in silico* generated mutants lacking negatively charged regions from various diverse *Neisseria* LbpBs, indicating that the location of the negatively charged regions is conserved within the *Neisseria spp.*

Using the structural model for LbpB lacking the negatively charged regions as a template, a structural model for wild-type MC58 was generated primarily to visualize the relative size of these regions (Figure 2, Panels B and C). Although there may be considerable uncertainty regarding the conformation and orientation of these two negatively charged regions, it seems evident that they could largely obscure the top surface of the C-lobe (illustrated in Figure 2 Panel C) and provide a considerable surface of interaction for interacting with cationic molecules. The core structural features of the N-lobe and C-lobe predicted in the models are almost certainly correct, as the structure of the N-lobe from *M. bovis* readily demonstrates [17], but the interaction between the lobes and their relative orientation will require experimental validation.

### The Negatively Charged Regions of LbpB are Responsible for Protection from Lactoferricin

**Figure 3:**
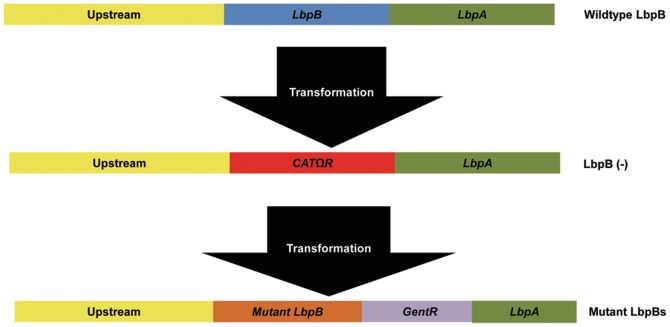
Scheme for generation of *N. meningitidis* LbpB mutant strains. PCR products of the indicated regions were amplified from plasmids isolated from yeast and used to transform *N. meningitidis* strain MC58 or its derivatives. The indicated regions were assembled in the yeast shuttle vector pYES2 (invitrogen) using the yeast homologous recombination method (Shao et al, 2009). In the first step, the *lbpB* gene is replaced by a chloramphenicol resistance gene. The resulting strain (N364) was transformed with PCR products that introduced wild-type (strain N368) or mutant *lbpB* genes (−LG or –LG, -SM; strains N365 and N366) followed by a gentamicin resistance cassette.

**Figure 4:**
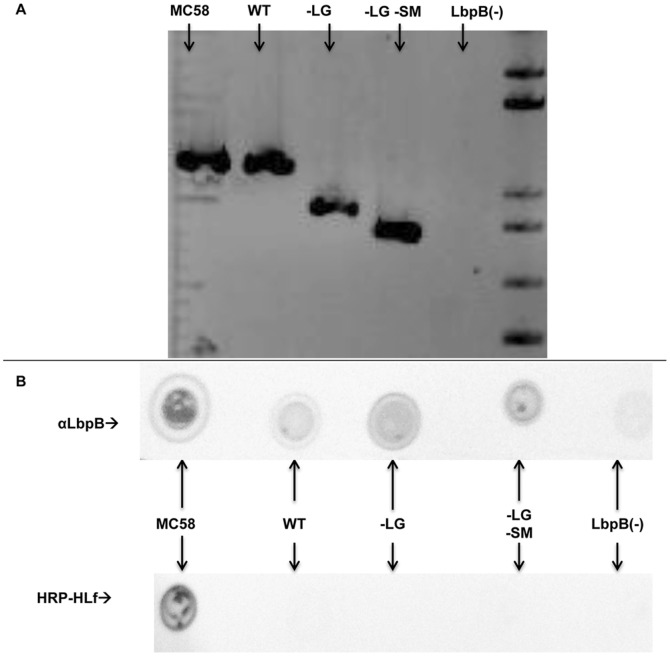
Characterization of the LbpB mutants. Panel A: Colony PCR was used to amplify the DNA region encoding the LbpB C-Lobe from wild-type and mutant *N. meningitidis* strains. PCR products were run on a 1.4% agarose gel with a 1KB plus DNA ladder (L) and visualized using ethidium bromide. Panel B: Solid phase binding assays were performed using MC58 LbpB specific antibodies (top) or HRP conjugated human lactoferrin (bottom). Antibodies were used to confirm the expression of LbpB at the cell surface, while human lactoferrin was used to evaluate LbpA expression. All strains except MC58 have an antibiotic resistance cassette inserted in front of the *lbpA* gene. In both panels MC58 is the parental wildtype *N. meningitidis* strain and WT indicates the gentamicin resistant strain that expresses the wild-type MC58 LbpB (N368). -LG indicates the strain expressing LbpB lacking the large negatively charged region (N365), and -LG –SM indicates the strain expressing LbpB that lack both negatively charged region (N366). LbpB(−) indicates the chloramphenicol resistant mutant lacking the *lbpB* gene (N364).

To explore the importance of the negatively charged regions in LbpB function, the mutant genes lacking the regions were transformed into *N. meningitidis* strain MC58. This was accomplished using a two-step approach in which *lbpB* was replaced with an chloramphenicol resistance cassette resulting in an LbpB deletion mutant (Figure 3) and subsequently mutant *lbpB* genes were used to replace the chloramphenicol cassette in a subsequent transformation step. Gentamicin resistance, due to a downstream gentamicin resistance cassette, was used to select for the mutant *lbpB* genes and a wild-type gene for the control strain (Figure 3). Assembly of the transforming DNA was performed in yeast using overlapping primers to restore the native leader peptide and anchor peptide region of LbpB and insert the *lbp* genes upstream of the gentamicin resistance gene. Colony PCR was used to confirm the presence of the *lbpB* genes in the wild-type and mutant strains (Figure 4, Panel A). The expression of the wild-type and mutant proteins at the cell surface was confirmed with solid phase binding assays (Figure 4, Panel B).

**Figure 5:**
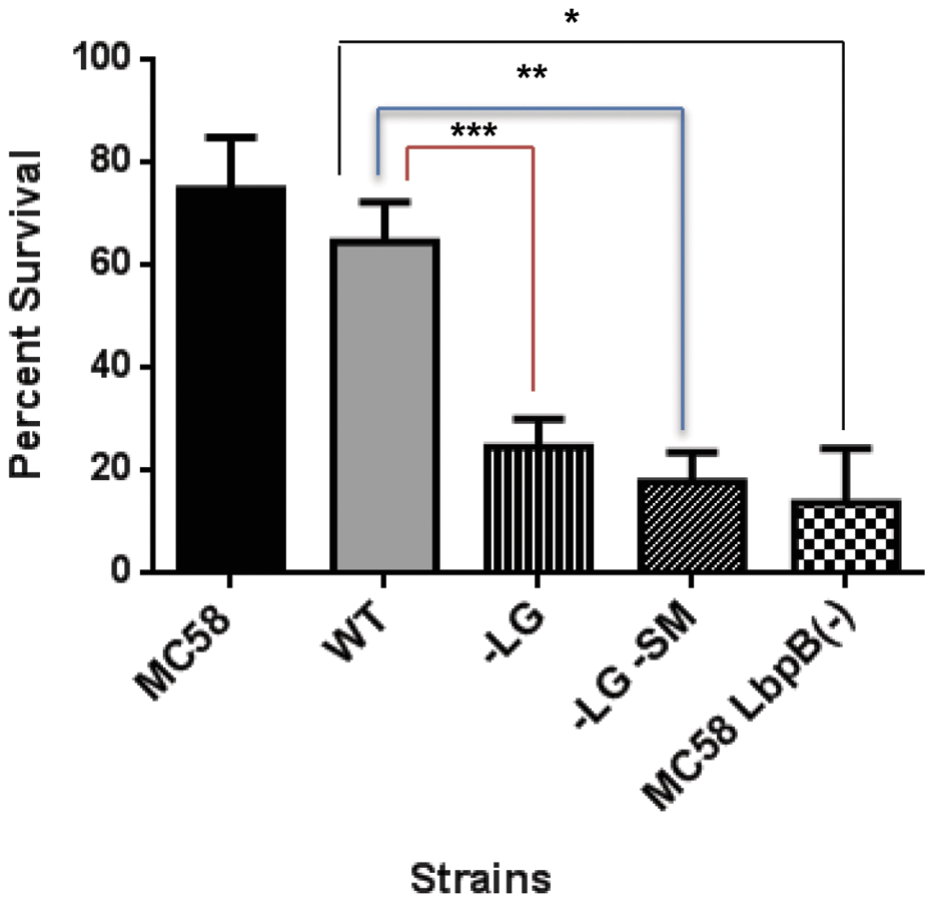
Lactoferricin killing assays with LbpB mutant strains . The parent MC58 strain (black bar) and intermediate chloramphenicol resistant LbpB-ve strain (N364, cross hatch bars) served as controls for strains with genes for the wild-type (N368, grey bar) and mutant (N365, vertical and N366, slanted bars) *lbpB* genes introduced. Bacterial survival was evaluated following treatment with 100 µM lactoferricin and normalized using percent survival. *, **, and *** indicate p values of 0.0052, 0.0069 and 0.0321 respectively. Each bar represents the average of a minimum of four replicates. Error bars represent the standard deviation of the mean for each sample. P values were calculated using the Tukey’s Multiple Comparison post hock test following a 1 Way Anova, performed using GraphPad Prism version 6.

To determine whether the negatively charged regions conferred resistance to the cationic antimicrobial peptide, lactoferricin, the strains derived from *N. meningitidis* MC58 expressing the wild-type and mutant LbpBs were treated with human lactoferricin and assessed for survival in killing assays. As expected, the strain expressing the wild-type LbpB was substantially protected from killing by lactoferricin relative to the strain lacking LbpB (Figure 5). In contrast the strains expressing mutant LbpBs lacking the large negatively charged region were susceptible to killing by lactoferricin. It is perhaps surprising that under these experimental conditions that the strain expressing LbpB with the small negatively charged region present (vertical bars, N365, Figure 5) was not protected against killing by lactoferricin.

### The Impact of NalP on Lactoferricin Protection

**Figure 6:**
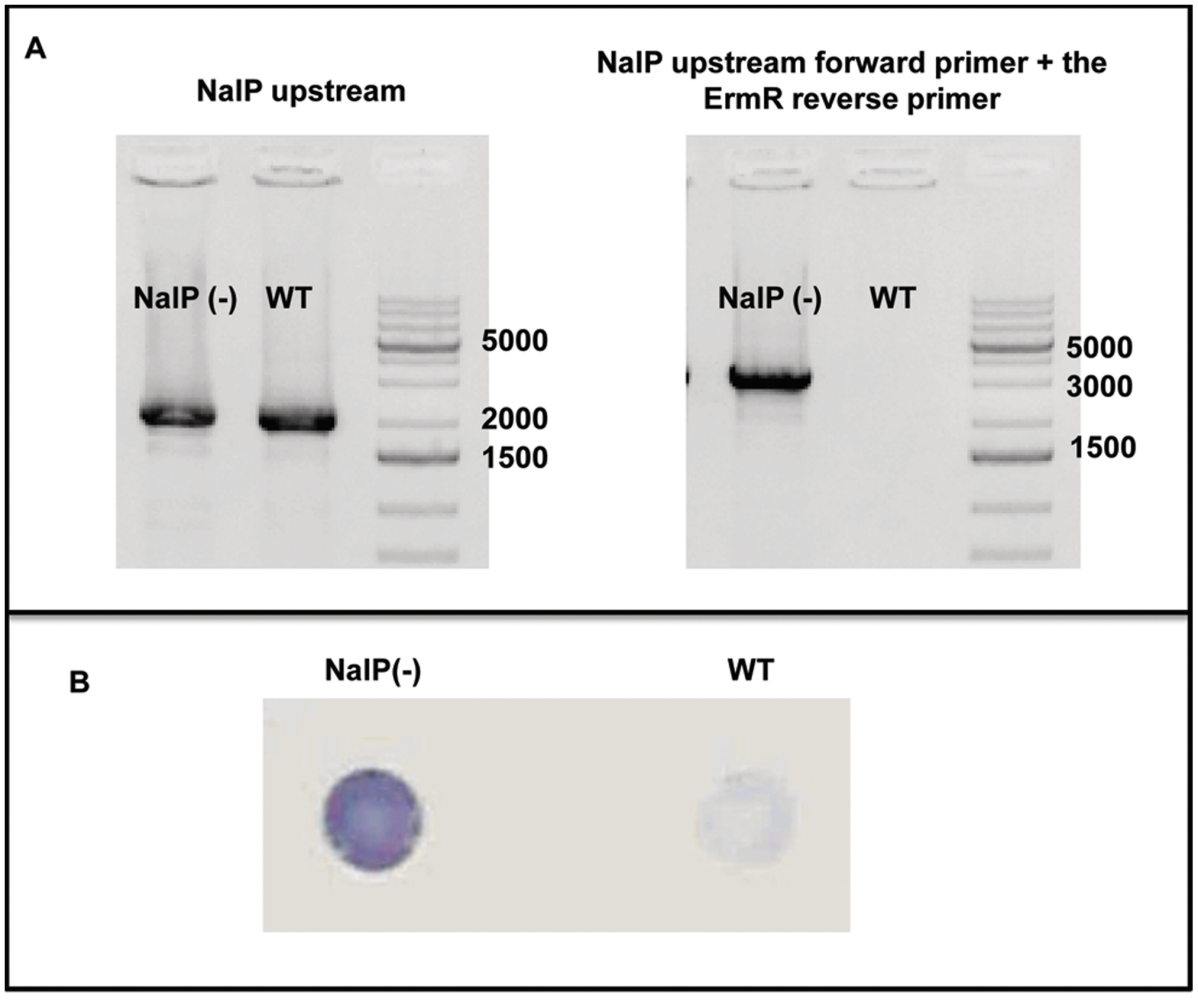
Characterization of NalP inactivation in MC58 NalP::ErmR mutants . A: Colony PCR was performed to confirm the replacement of the initial kB of the *nalP* gene with an erythromycin resistance cassette using the wild-type parental *N. meningitidis* MC58 strain as a control (WT). Colony PCR was performed using primers for the NalP upstream region, and the NalP upstream region with the erythromycin resistance cassette. B: Solid phase binding assays were performed using LbpB specific antibodies to confirm that inactivation of NalP resulted in a significant reduction of LbpB release from the cell surface.

LbpB is released from the cell surface by the autotransporter NalP, through proteolytic cleavage in the anchor peptide region of LbpB [14]. Thus the released LbpB retains the negatively charged regions. As our killing assay with lactoferricin includes a wash step prior to exposure to lactoferricin, released LbpB is likely lost and therefore unable to contribute to lactoferricin protection. To explore the impact of NalP on LbpB-mediated protection, a *N. meiningitidis* MC58 NalP mutant was prepared by replacing the first kilobase of the *nalP* gene with an erythromycin resistance cassette. The placement of the erythromycin resistance cassette in the *N. meningitidis* NalP(−) strains was confirmed using colony PCR and solid phase binding assays (Figure 6).

**Figure 7:**
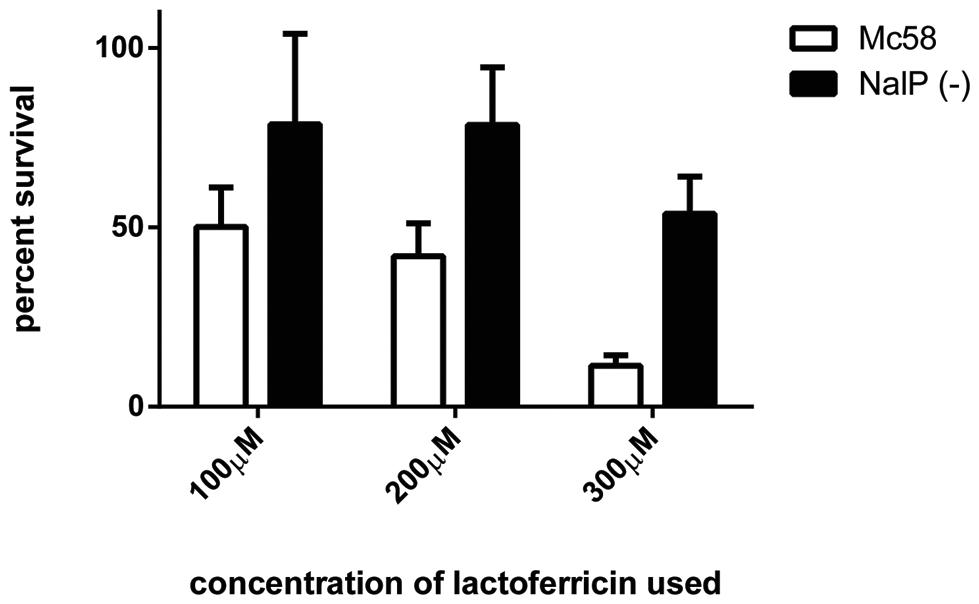
The impact of NalP on LbpB-mediated lactoferricin protection. Lactoferricin killing assays were performed by exposing strains to various concentrations of lactoferricin (x axis) and enumerating bacterial survival by plating. Results were normalized by converting bacterial survival to percent survival (y axis). Bars represent the average of five experiments with outliers removed using the grubs outlier test with an alpha value of 0.05. Error bars represent the standard error of the mean for each sample. A two way Anova was performed using GraphPad Prism Version 6 to determine the significance of the concentration of lactoferricin and the presence of a functional NalP (p-value of 0.0083).

A killing assay with varying levels of lactoferricin was performed comparing the NalP deficient mutant to the wild-type strain (Figure 7). The increased level of cell-associated LbpB in the NalP (−) strain (Figure 6 B) is anticipated to better mimic the level of LbpB *in vivo* as released LbpB would likely be available to locally complex with cationic peptides under most *in vivo* conditions. The results clearly show that the NalP deficient mutant provides superior protection against lactoferricin, particularly at higher concentrations of lactoferricin, (compare black and white bars in Figure 7).

**Figure 8:**
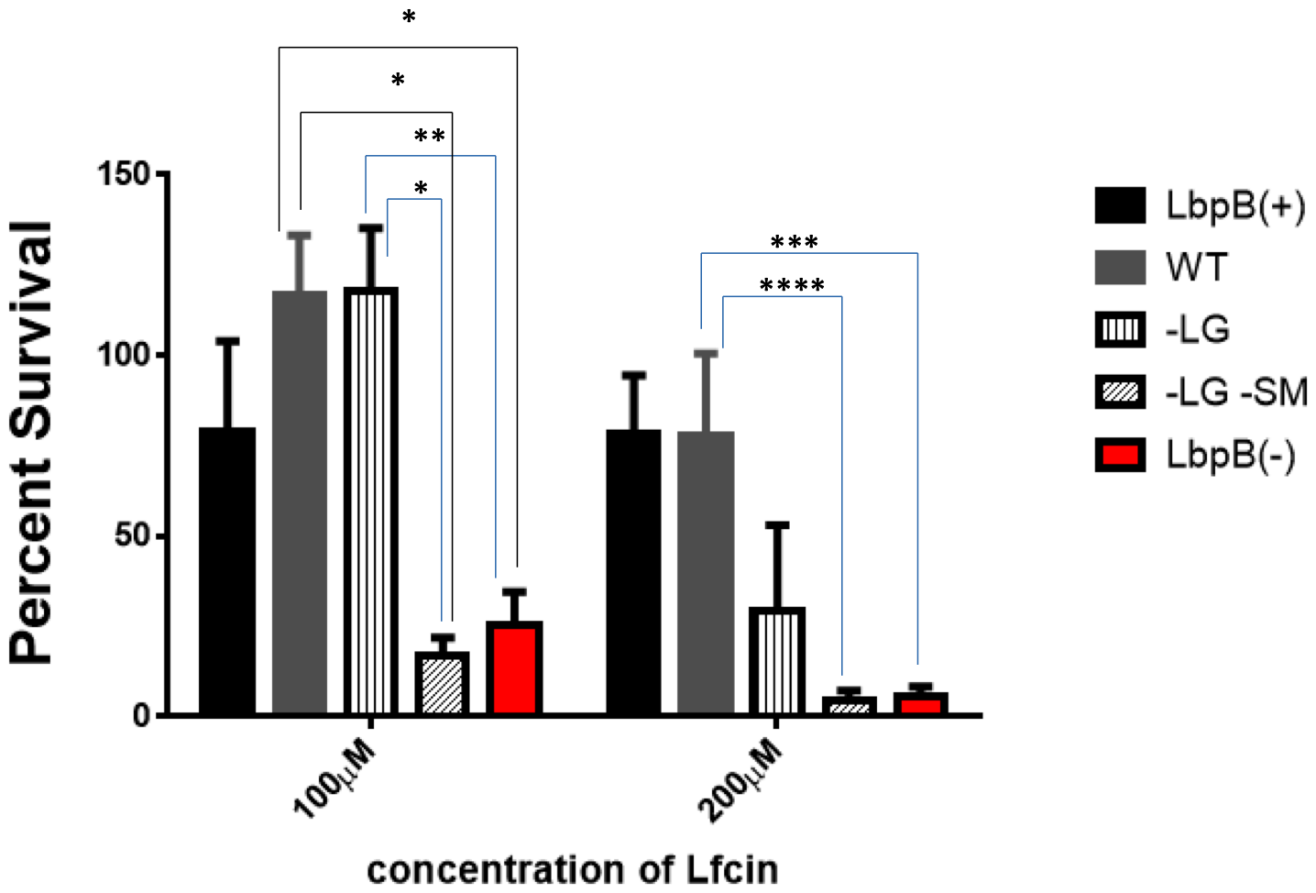
Lactoferricin killing assays with LbpB mutant strains in NalP defincent backgound . The parent LbpB(+) NalP(−) strain (black bar) and intermediate chloramphenicol resistant LbpB(−) NalP(−) strain (red bars) served as controls for strains with genes for the wild-type (grey bar) and mutant (−LG vertical and -LG -SM slanted bars) *lbpB* genes introduced. Bacterial survival was evaluated following treatment with 100 µM lactoferricin and normalized using percent survival. *, **, ***, and **** indicate p values of <0.01, 0.0125 0.0176 and 0.03 respectively. Each bar represents the average of a minimum of four replicates. Error bars represent the standard deviation of the mean for each sample. P values were calculated using the Tukey’s Multiple Comparison post hock test following a 1 Way Anova, performed using GraphPad Prism version 6.

In view of the superior protection provided by the NalP deficient mutant, the protection conferred by the individual negatively charged regions was reexamined using a NalP deficient background. The results demonstrate that in the absence of cleavage by NalP, the presence of both the small and large negatively charged regions provided considerable protection against lactoferricin, particularly at lower concentrations (vertical bars, Figure 8). Thus at 100 µM lactoferrin, LbpB containing only the small negatively charged region (−LG, vertical bars) was as protective as the wild-type LbpB containing both negatively charged regions. At 200 µM lactoferricin the protection conferred by the LbpB containing only the small negatively charged region was considerably less than the wild-type LbpB. It is likely that these experiments in the NalP deficient strain is a better representation of most *in vivo* conditions as the removal of released LbpB during our wash steps in the killing assay reduces the levels of LbpB available to interact with lactoferricin more than would be expected *in vivo.*

### The Importance of the Bacterial Capsule

**Figure 9:**
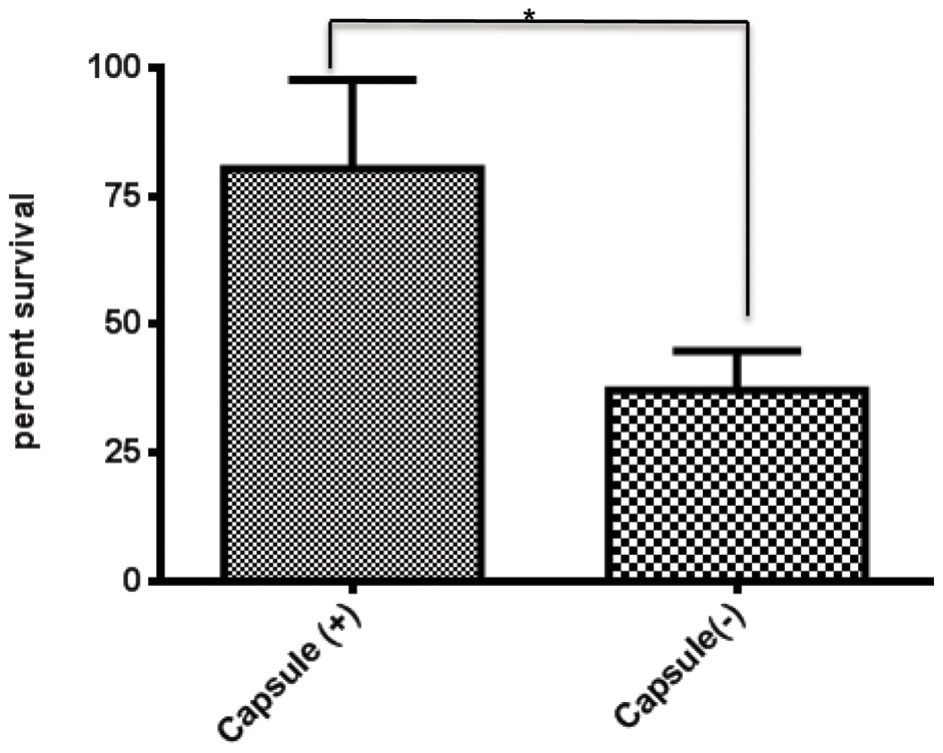
The impact of the bacterial capsule on lactoferricin mediated protection . A: Lactoferricin killing assays were performed with 100 µM of lactoferricin on a capsule deficient *N. meningitidis* MC58 strain (capsule(−)) and a *N. meningitidis* wildtype MC58 strain (capsule(+)). Bacteria were treated with lactoferricin for one hour before being plated to enumerate bacterial survival that was normalized using percent survival. Each bar represents the average of 4 experiments, while error bars represent the standard deviation of the mean. * indicates a p value of 0.0638.

Although the mode of action of lactoferricin against bacteria has yet to be determined, current models suggest that lactoferricin disrupts the cell membrane, resulting in bacterial death. In encapsulated organisms it has been suggested that the polysaccharide capsule acts as a barrier between the cell membrane and lactoferricin, and the negatively charged sialic acid present in the dominant *N. meningitidis* capsule types, would enhance the protective effect of the bacterial capsule. In this study we utilized a MC58 strain with an inactivated *siaD* gene to evaluate the impact of the bacterial capsule on lactoferricin protection. The *siaD* mutant has been previously shown to disrupt capsule synthesis in *N. meningitidis*. The mutation in the strain used in this study was confirmed using PCR, capsule type B specific antibodies and electron microscopy [21]. The capsule was found to confer moderate protection in the lactoferricin killing assay (p value of 0.06), at a concentration of 100 µM lactoferricin (Figure 9).

## Discussion

In a previous study it was shown that LbpB and not LbpA conferred protection against the cationic antimicrobial peptide, lactoferricin, and was postulated that this might be due to the relatively large clusters of negatively charged amino acids present in C-terminal lobe of the LbpB [10]. Fortunately the removal of the negatively charged regions from the *N. meningitidis* MC58 LbpB did not impact the stability of the protein (Figure 1), or its export to the cell surface (Figure 4B), thus enabling us to determine whether or not these regions contributed to protection against the killing activity of lactoferricin on meningococcal cells. In the killing assays it was shown that removal of the negatively charged regions virtually abolished the protection against lactoferricin (Figure 8). These results clearly provide support for the role of the negatively charged regions in protection against lactoferricin, and the invariable presence of the large negatively charged region in LbpBs from strains of *N. meningitidis* [11] suggest that this is an important function *in vivo.*

Considering the size of the ‘small’ negatively charged region (30 amino acids, Figure 2) and its presence in 2/3 of *N. meningitidis* isolates, its limited impact on the killing by lactoferricin in our first set of assays was somewhat surprising (Figure 5). However, it is important to note the relatively low level of LbpB remaining associated with the cell surface in the wild-type strain expressing NalP (Figure 6B), as this is the only LbpB that would be present when the cells are exposed to lactoferricin in the killing assay. It seems likely that the extensive washing that removed any released LbpB for our killing assays does not truly reflect the situation under most *in vivo* conditions. The release of LbpB by NalP cleavage may not preclude LbpB from acting locally at the site of infection or inflammation, thus released LbpB could contribute to protection against lactoferricin or other cationic peptides. Although the use of a NalP mutant to retain all of the LbpB at the cell surface (Figure 6B) may overestimate its role *in vivo*, the experiments with NalP mutants do compensate for the artificial washing conditions used for our killing assay. Thus the demonstration that the small negatively charged region can provide significant protection against lactoferricin in an NalP mutant (−LG, vertical bars, Figure 8), suggests that the presence of this cluster of negatively charged amino acids confers additional protection against cationic peptides *in vivo.*

Similar to TbpB [18], LbpB is a relatively large surface lipoprotein capable of extending a substantial distance from the outer membrane surface, thus would be accessible to many host proteins, including immunoglobulins. Analysis of convalescent-phase immune sera from patients with *Moraxella catarrhalis* pulmonary infection revealed that the immune response against LbpB and TbpB constitutes a substantial portion of the total response against *M. catarrhalis* [22]. The fairly extensive negatively charged regions (Figure 2) could also serve as a target for the host immune response and might account for some of the cross-reactivity observed against meningococcal LbpB in convalescent patient sera [23]. The selective cleavage and release of LbpB by the phase variable autotransporter protein, NalP, provides a mechanism for evading the adaptive immune response, as it improved bacterial viability during serum bactericidal assays with anti-LbpB antisera [14]. The fact that TbpB is not released by NalP, although it clearly is a target for bactericidal antibodies, suggests that it plays a more critical role in iron acquisition than LbpB. Thus it is possible that the acquisition of negatively charged regions by LbpB to counteract the action of cationic antimicrobial peptides coincided with a reduction in its role in iron acquisition from lactoferrin.

The role of LbpB in iron acquisition is uncertain as earlier studies demonstrating a loss in growth dependent upon exogenous lactoferrin by insertional activation of the *lbpB* gene [8], [9] also affected the expression of the downstream *lbpA* gene. Evaluating the role of LbpB in iron acquisition is further complicated by the activity of NalP which releases LbpB from the cell surface [14]. Although it has been suggested released LbpB may act in a similar fashion to the HasA hemophore, facilitating the capture of lactoferrin by LbpA [14], this would rely on an enhanced affinity of LbpB-bound lactoferrin for LbpA, and likely would be much less effective than the membrane bound form. In this context it is important to consider that formation of a ternary complex between TbpA, TbpB and transferrin was dependent upon the presence of the intact anchor peptide [24], and that the intact anchor peptide would be absent from the released form of LbpB. An interesting possibility is that the phase variable expression of NalP could modulate the role of LbpB in iron transport and immune evasion, but should be supported by definitive experiments demonstrating that cell bound LbpB can assist in the acquisition of iron from human lactoferrin.

The polysaccharide capsule is commonly considered an important virulence factor for many bacterial pathogens as it inhibits opsonophagocytosis and other host defense mechanisms by multiple mechanisms [25]. The negatively charged capsules common to many *N. meningitidis* strains would be expected to provide some protection against cationic antimicrobial peptides (CAPs), such as lactoferricin, that are thought to act on a cell by disrupting the bacterial membrane. Our results confirm that the polysaccharide capsule does provide some protection against lactoferricin (Figure 9). It is important to note that the limited amount of LbpB present in this experiment potentially overestimates the contributions of the capsule to lactoferricin protection. Since the strains used in this experiment are wild-type for both LbpB and NalP, there is significant removal of LbpB during washing steps (Figure 6B) and the extent to which the limited amount of LbpB would enhance the level of protection in the wild-type and capsule-deficient strains is uncertain. Notably the results in Figure 5 demonstrate that the residual protection in a LbpB deficient mutant, presumably due primarily to capsule was only 20% (compared to 65% in the presence of LbpB), and this was performed in a wild-type NalP background. Considering the loss of LbpB during washing steps, the results suggest that LbpB likely plays a greater role than capsule in protection from cationic peptides *in vivo.*

The importance of lactoferrin receptors *in vivo* has been best demonstrated in the *N. gonorrhoeae* male human urogenital infection model [6]. Co-infection of male subjects, with *N. gonorrhoeae* FA1090 (a naturally LbpA/B deficient strain) and an isogenic mutant expressing functional Lbps, demonstrated a competitive advantage conferred by the presence of the Lbp receptors *in vivo*. Since the Tbp receptor proteins were shown to be even more important for survival in this model, it was assumed that the advantage by strains expressing Lbps was conferred by greater access to iron at the mucosal surface. It would be interesting to perform experiments in a relevant model that would investigate the role of the individual receptor proteins in survival, and use mutants deficient in the negatively charged regions to evaluate the role of protection against cationic peptides.

We suspect that the negatively charged regions evolved in order to combat the high concentration of lactoferricin and other cationic peptides at sites of inflammation thereby reducing bacterial susceptibility. The proposed immunodominance of these regions could explain the high proportion of anti-LbpB antibodies in convalescent patient sera and the NalP mediated release of LbpB. Released LbpB would retain its negatively charged regions and therefore remove lactoferricin and bactericidal antibodies from circulation before it can impact the bacteria. Further study is required to determine the specificity of LbpB mediated protection as the negatively charged regions are likely to confer protection against other antimicrobial peptides encountered during the course of an infection.

## Materials and Methods

### Bacterial Strains

The strains of *N. meningitidis* used in the killing assays are listed in Table 1. Each strain was streaked onto chocolate or brain heart infusion (BHI) agar plates and grown at 37°C with 5% CO2. Liquid cultures were inoculated by resuspending isolated colonies from overnight plates in BHI and then diluting to an A600 of 0.01–0.05 in BHI broth containing 100 µmol/L Desferal (an iron chelator to induce expression of LbpB) and grown to an A600 of 0.1–0.2.

**Table 1:** Neisseria meningitidis strains.

Strain #	Derived From	Phenotype	Reference
N360		Wildtype, strain MC58	[28]
N364	N360	LbpB(−), chloramphenicol resistant	This study
N365	N364	LbpB lacking the large negatively charged region (+), gentamicin resistant	This study
N366	N364	LbpB lacking both negatively charged regions, gentamicin resistant	This study
N368	N364	Wildtype LbpB, gentamicin resistant	This study
N367	N360	NalP (−), Erythromycin resistant	This study
N369	N364	NalP(−) LbpB(−) Chloramphenicol and Erythromycin resistant	This study
N375	N366	NalP(−) LbpB lacking both negatively charged regions, Erythromycin and gentamicin resistant	This study
N376	N365	NalP(−) LbpB lacking the large negatively charged region (+), Erythromycin and gentamicin resistant	This study
N377	N368	NalP(−) Wildtype LbpB Erythromycin and gentamicin resistant	This study

### LbpB Recombinant Expression

Primers O3319 and O2774 (Table 2) were used to amplify a segment of the *lbpB* gene encoding amino acids 54 to 737 of LbpB from *N. meningitidis* strain MC58 genomic DNA for cloning into a custom vector expression vector [17]. Primers O3131 and O3130 were used in inverse PCR to excise the large negatively charged region while primers O3129 and O3128 were used to remove the small negatively charged region. The PCR products were self-ligated after phosphorylation. After expression of the recombinant Mbp::LbpB fusion proteins with autoinduction media, the cells were lysed using a homogenizer and the polyhistidine tagged recombinant fusion proteins were purified using Ni-NTA chromatography.

**Table 2:** Primers used for amplifying segments of the MC58 *lbpB* gene.

Oligo	Sequence	Description
O3319	GCGGATCCCCAAGGCGGAATATTGCTTC	Forward primer with BamH1 site preceding aa 54 of mature LbpB
O2774	CTTACTCTAGACTCATTTTTCCACCTCCTGCATATC	Reverse primer with Xba site after stop codon of mature LbpB
O3131	TCAGACGGCATCCTGCCC	Forward primer to region encoding aa 534–539 (−LG)
O3130	CGCCTTCGGTTTGGCGGC	Reverse primer to region encoding aa 463–468 (−LG)
O3129	TTCGGCGTGGTATTCGGTG	Forward primer to region encoding aa 722–727 (−SM)
O3128	AGTTATACCAAGAGATTTCCCATC	Reverse primer to to region encoding aa 682–690 (−SM)

### Yeast DNA Assembler Method

DNA constructs for generating the LbpB and NalP mutations were assembled via a previously described yeast homologous recombination system [26]. In brief, DNA fragments to be assembled were amplified using Phusion polymerase (New England Bioscience) with overlapping homologous regions of at least 50bps. All fragments, including the yeast-*E. coli* shuttle vector pYES2 (Invitrogen) which functioned as a plasmid backbone for assembly, were electroporated into a *S. cervesiae* strain deficient in uracil synthesis. The shuttle vector contained *ura3* allowing identification of successful recombination events by uracil sensitivity on YNB agar plates. Selected colonies were restreaked on YNB agar and plasmids were isolated using a EZNA yeast plasmid isolation kit (Omega Biotek cat No. D3376). The plasmid preparations were used directly for PCR amplification required for generating sufficient DNA for natural transformation of *N. meningitidis.*

### Transformation of *N. meningitidis*

50–100 µl of a suspension of *N. meningitidis* cells grown on BHI or chocolate agar were mixed with 200 ng of linear PCR product and spotted onto a chocolate or BHI agar plate and allowed to dry. Plates were incubated for a minimum of 4 to a maximum of 24 hours at 37°C +5%CO_2_. Bacteria were scraped from plates and spread onto BHI agar plates containing the appropriate antibiotics for selection (10 µg/ml chloramphenicol, 15 µg/ml gentamicin, 5 µg/ml erythromycin).

### Solid Phase Binding Assays

High stringency solid phase binding assays, as previously described, were used as an additional step in confirming LbpB, and NalP mutants [7]. The assays were modified for the use of polyclonal LbpB rabbit antibodies in place of HRP conjugated lactoferrin or transferrin. In brief, suspended bacteria were spotted onto ME-nitrocellulose membrane and allowed to dry before blocking with 2% skim milk in TBS buffer (W/V). Following 15–30 minute membranes were incubated in TBS containing αLbpB rabbit sera, for 2–4 hours. Membranes were then washed in TBS and subjected to a second blocking step before being incubated with HRP conjugated goat αRabbit antibodies. Membranes were finally washed in TBS and then developed with HRP color development reagent.

### Killing Assays

Killing assays were performed as previously described [27] using a human lactoferricin-derived peptide, LfcinH (1–11), comprising the initial 11 amino acids at the N-terminus of human lactoferrin (GRRRRSVQWCA) that was synthesized by CanPeptide (Montreal, Canada). There were no chemical modifications of the C-terminus, N-terminus, or amino acid side groups in the peptide. Briefly, bacteria were grown as previously described to an OD of 0.1–0.2 (A600) and resuspended in AS solution (150 mmol/L NaCl, 1 mmol/L MgCl2, 50 µmol/L CaCl2 and 1 mmol/L K2PO4, pH 7.2) with and without 100 µmol/L LfcinH (1–11). Bacteria were incubated in AS solution for 1 h and then plated using a 1 in 5 serial dilution on BHI or TH plates. The plates with incubated overnight at 37°C with 5% CO2 and enumerated the next day to determine CFU.

### Statistical Analyses

For killing assays, percent bacterial survival was determined by dividing the CFU/ml of bacteria incubated with lactoferricin by the CFU/ml of those incubated in AS only. The data was analyzed with Graphpad Prism version 6 software, a one or two -way analyses of variance (ANOVA) were used where necessary. Alternatively, T tests were used to determine significance of the difference in survival between the wild-type *N. meningitidis* MC58 strain and an isogenic capsule mutant. Experiments were replicated on multiple days producing a minimum of three replicates for each strain. Any outliers were removed using the Grubbs outlier test with α set to 0.01.

### Structural Models

Structural models for LbpBs were developed with the Swiss Model server (http://swissmodel.expasy.org) using the available structures for TbpB from *A. pleuropneumonieae* [18] or *N. meningitidis* M982 [19] as a starting template. PyMol software (http://www.pymol.org) was used to visualize and annotate the models.
